# Retrospective Study of 18 Titanium Alloy Crowns Produced by Computer-Aided Design and Manufacturing in Dogs

**DOI:** 10.3389/fvets.2019.00097

**Published:** 2019-04-05

**Authors:** Lisa A. Mestrinho, Inês Gordo, Jerzy Gawor, Nuno Leal, Maria Niza

**Affiliations:** ^1^CIISA-Centre for Interdisciplinary Research in Animal Health, Faculty of Veterinary Medicine, University of Lisbon, Lisbon, Portugal; ^2^Pride Veterinary Centre, Derby, United Kingdom; ^3^Klinika Weterynaryjna Arka, Krakow, Poland; ^4^School of Dental Medicine, University of Lisbon, Lisbon, Portugal

**Keywords:** full veneer crown, prosthodontics, crown preparation, computer-aided design, computer-aided manufacturing, titanium alloy, dog

## Abstract

Computer-aided design (CAD) and computer-aided manufacturing (CAM) technology is routinely used in various fields of human dentistry, particularly prosthodontics. Reverse engineering and additive manufacturing allow the technician to create an easier, faster and more accurate restoration, with a natural design and adequate strength. Eighteen titanium alloy full crowns were produced for canine teeth of 7 working dogs using CAD/CAM technology (3D BioCare, Nobel Biocare). Reasons for crown therapy included abrasion, enamel infraction, and crown fracture. Crown preparation was routinely performed, and impressions were delivered to the laboratory where digital impressions were performed with a lab scanner. Using 3D dental design software, the metal crown was designed and sent for manufacture. Each prosthodontic crown was then carved from a solid titanium alloy block to obtain the final crown. All prosthodontic crowns were an adequate fit, and cementation was routinely performed. Crowns were lost from 2 canine teeth during the dogs' normal working activity, in one case, for 2 times. In all cases, replicas were requested. In the first case, the second cementation was successful. In the second case, the second crown was again lost and only the third cementation was successful. Follow up of all cases range from 12 to 62 months. Mean survival time for the crowns was 58.0 months. Here, CAD/CAM technology is shown to be a useful tool for manufacturing accurate prosthodontic crowns for veterinary patients. Moreover, CAD/CAM enables the production of prosthodontic crown replicas in a very short time and at relatively low cost compared to traditional methods, consequently eliminating the need for at least one anesthetic procedure in the incidence of crown cementation failure.

## Introduction

Working dogs are generally more prone to dental damage related to trauma such as wear, enamel infraction, and fractures (1). Canine tooth fractures are frequently reported in working dogs, possibly due to their anatomic functions and exposure (2–4).

Dental fractures with pulp exposure can lead to pulp necrosis, which is associated with pain and, eventually, periapical disease. Endodontic therapy or tooth extraction are possible strategies in such cases, but only root canal treatment allows maintenance of the tooth function and aesthetics, and root canal treatment is also less invasive than tooth extraction (1, 5).

Crown therapy can help to maintain the function of a tooth after endodontic procedures and to prevent wear and further decay of teeth in cases of enamel infraction (1, 6). The most common reasons evoked against the use of crown therapy in veterinary dentistry include economic constraints, the risk of weakening the tooth during the crown preparation process and the need for multiple sedation sessions under general anesthesia during the crown-building process. The increased awareness of the importance of maintaining functional teeth in working dogs make them good candidates for crown therapy in veterinary medicine, as it can improve the outcomes of routine dental restoration and decrease the incidence of endodontic pathology (6).

Compared with full ceramic or metal-ceramic alloys, metal crowns are potentially more resistant and durable, needing the least removal of tooth material at time of crown preparation. In veterinary medicine, the economic factor often plays a more important role than the aesthetic one, which makes metal crowns a very attractive option. There are clinical studies and case reports reporting the use of gold, iron, and alloys of cobalt, chrome, molybdenum, nickel and others non-precious nickel free alloys in veterinary medicine (6–9).

Computer-aided design (CAD) and computer-aided manufacturing (CAM) technology has been validated as an equally appropriated method for crown manufacturing, in the human dentistry field (10–12). Reverse engineering and additive manufacturing allow the technician to create an easier, faster and more accurate restoration, with a better marginal adaptation, natural design, and adequate strength (10–12).

The purpose of the current study was to determine the applicability and effectiveness of full-veneer crowns made with CAD/CAM technology for working dogs. Another aim was to evaluate the resistance to, acceptance of and possible complications related to the use of titanium alloy crowns in dogs. To the knowledge of the authors, there are currently no published data on the use of CAD/CAM for crown shape development in a clinical veterinary dentistry setting.

## Materials and Methods

In this clinical study, 18 titanium alloy crowns (NobelProcera Scan and Design Services, Nobel Biocare) were placed in the canine teeth of seven working dogs with teeth fractures, enamel infraction or significant abrasion. All animals were seen on consultation and anesthetized for oral examination and treatment. Dental radiographs (Vitascan, Duerr Dental) of the involved teeth were taken before crown preparation.

The anesthetic protocol included sedation with dexmedetomidine 5 μg/kg (Dexdomitor, Esteve) and butorphanol 0.2 mg/kg (Butomidor, Richter Pharma AG) intravenously, induction with propofol 1–2 mg/kg to effect (Propovet, Ecuphar), with diazepam 0.2 mg/kg (Diazepam, Labesfal) and maintenance with isoflurane (Isoflo, Ecuphar) and oxygen.

Teeth diagnosed with endodontic disease were treated prior to the crown therapy. Crown preparation with a circumferential supragingival (1–2 mm) chamfer margin was made using a round number 1 diamond burr (A1 ISO 018, R&S) and a tronco-conical diamond burr (6856 ISO 018, Komet). Then, individual impressions of the canine teeth were taken using a combination of heavy and light vinyl polysiloxane (Hydrosoft Putty Fast Set and Hydrosoft Light Fast Set, R&S). Alginate (Turboprint, R&S) full mouth impressions and bite registrations using bite wax (Cera, Reus) were also made. Stone models were generated after the animal recovered from anesthesia, and impressions were kept moist during that time.

All dogs were discharged on the same day of the procedure with no temporary crown but with recommendations to suspend working activities as well as chewing hard objects while waiting for the prosthodontic crown to be placed.

**Figure 1:**
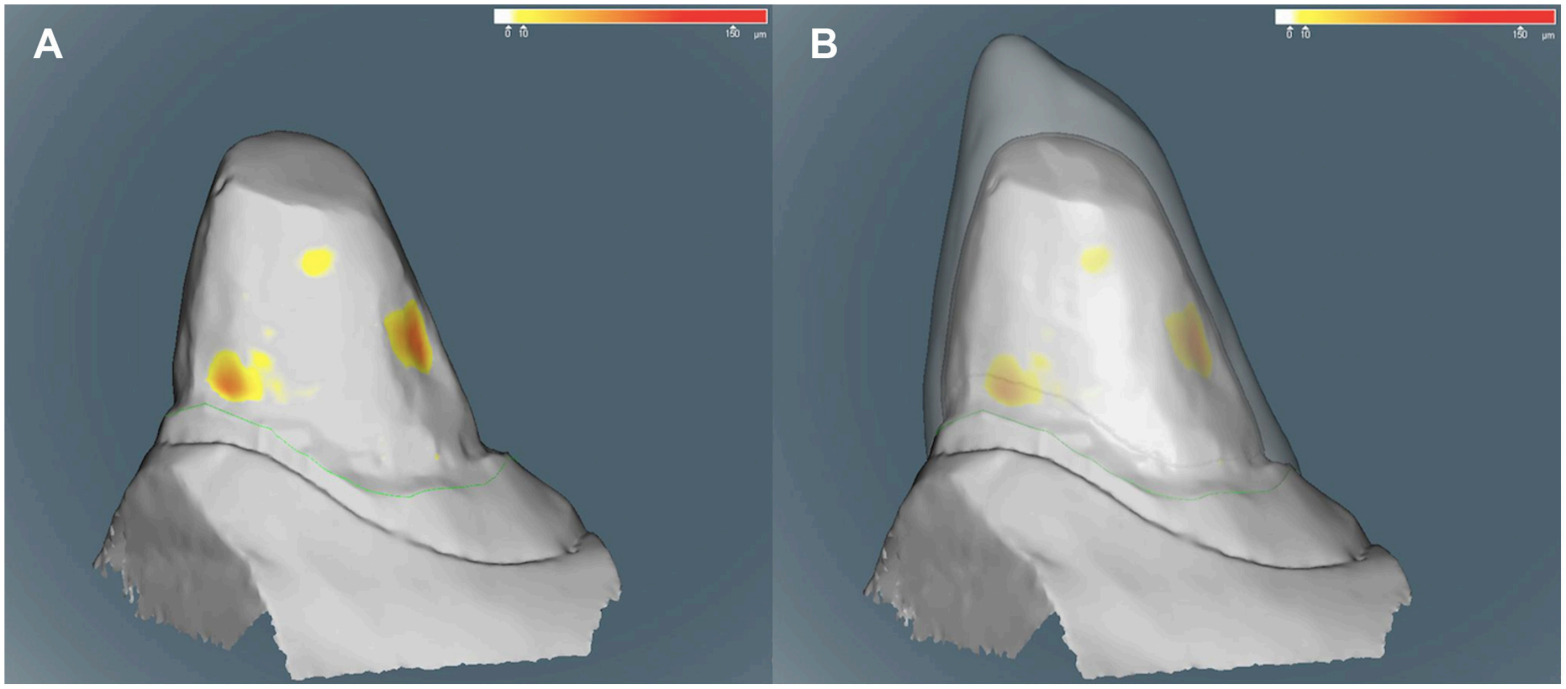
**(A,B)** Computer images during the design of one crown. The heat map indicates the depth (in μm) of areas considered retentive. These yellow areas were used to guide the design using a real time feedback provided by the software. **(A)** The technician first sets the margins (green line). **(B)** A predefined crown with at least 0.4 mm is automatically generated (transparent contour). From this point thickness can be added evenly or just in some areas. Additionally, the inside contour of the crown can be adjusted to avoid the retentive areas and produce the best possible prosthodontic crown. Note the added thickness on the mesial side of the cusp **(B)**.

The crown designs were produced using CAD/CAM technology (3D BioCare, Nobel Biocare). The CAD was performed in the prosthesis laboratory by the authors and the CAM was outsourced (Nobel Biocare). The CAD started after the production of dental models with a scannable Type-IV gypsum (Zhermack Elite Rock). The models were digitized by using a scanner (Nobel Procera 2G). The information was then imported to the 3D dental design software (DTX Studio Design, version 1.7.8.6) which automatically produces an image. Since the metal crown has to fit into a predetermined titanium block of 80 × 80 × 30 mm, the software gives real-time feedback through a warning function, informing if the crown cannot be produced. The margin was the first to be identified, marked and set. Consequently, the software provides, automatically, a preset crown based on the contour of the scanned clinical crown. The minimum thickness of this predetermined shape is 0.4 mm. Again, using the real-time feedback, the final design is set by adding more thickness all around the crown or specifically in some areas, e.g., cusp (Figure 1). Information of the final crown is saved and sent to another laboratory (NobelProcera Laboratory), where the prosthodontic crown is milled from a solid titanium alloy grade 2 (titanium-aluminum-vanadium) block to obtain the final crown.

The cementation was routinely performed with resin dental cement (Panavia 21, Kuraray Dental) after cleaning the prepared teeth and applying 37% orthophosphoric acid etching gel (Octacid, Clarben). Previously, at the dental laboratory, crowns were sand blasted.

The dogs' working activities were resumed 24 h after cementing the definitive crowns, but with recommendations for a careful and gradual increase in work intensity during the first 2 weeks. The follow-up period was at least 1 year and included regular telephone-based updates and check-up appointment at 6 months and 1 year.

Survival probability of crown cementation was assessed using Kaplan-Meyer analysis. Cases which the crown was cemented at the end of the study were censored (= success) and cases of crown lost were considered events (= failure), regardless if a new cementation took place, it was not counted as another crown.

## Results

Eighteen titanium crowns produced using CAD/CAM were placed in 7 dogs: 5 working Belgian Shepherd, Malinois (3 females and 2 males), 1 male German Shepherd and 1 male Border Collie. Additionally, 3 crowns were applied as replacements for lost ones. The activities of working dogs included security (2), Mondioring (a sport) (3), and Agility (a sport) (2).

The mean age ±standard deviation (SD) at time of prosthodontic crown cementation was 2.4 ± 1.3 years and the mean weight was 28.5 ± 5.4 kg. Prior to crown therapy, in 4 of the 7 dogs, the owners reported behavior changes involving decreased performance and/or reluctance to work, all presented dental fractures. Three dogs received four crowns each; 2 crowns were cemented on to the mandibular canine teeth in 2 dogs; 1 crown was cemented on to the right maxillary canine tooth of 1 dog; and 1 crown was cemented on to the left mandibular canine tooth of the remaining dog. Indications for crown therapy were abrasion, enamel infraction, and fracture, detailed in Table 1. Crown therapy was performed no earlier than 3 months after endodontic treatment in the 3 cases of complicated crown fracture, after radiographic evidence of periapical recovery. In one dog, tooth #16, a crown lengthening procedure was also performed with the endodontic treatment.

**Table 1:** Summary information of 18 CAD/CAM processed prosthodontic crowns in seven dogs.

**No**.	**Tooth**	**Dog**	**Sex**	**Age (years)**	**Weight (kg)**	**Diagnosis**	**Months**	**Adjunct endodontic or periodontal treatment**	**Complication and approach**	**Activity**
1	304	1	F	2.17	28	CCF	62	RCT	None	Security
2	404	1	F	2.17	28	Ab	62	None	None	Security
3	104	2	F	2.2	28	Ab	58	None	None	Security
4	204	2	F	2.2	28	Ab	53	None	CL	Security
4	204	2	F	2.2	29.2	CL	5^*^	None	None	Security
5	304	2	F	2.2	28	Ab+EI	58	None	None	Security
6	404	2	F	2.2	28	Ab+EI	58	None	None	Security
7	104	3	F	1.1	32	Ab	54	None	None	Mondioring
8	204	3	F	1.1	32	Ab	54	None	None	Mondioring
9	304	3	F	1,2	32	Ab+EI	55	None	None	Mondioring
10	404	3	F	1,2	32	Ab+EI	55	None	None	Mondioring
11	104	4	M	3.67	31.5	Ab	50	None	None	Mondioring
12	204	4	M	3.67	31.5	Ab	50	None	None	Mondioring
13	304	4	M	3.67	31.5	Ab+EI	6	None	CL	Mondioring
13	304	4	M	4.17	31.5	CL	1^*^	None	CL; new crown prep with axial grooves	Mondioring
13	304	4	M	4.25	31.5	CL	43^*^	None	None	Mondioring
14	404	4	M	3.67	31.5	Ab+EI	50	None	None	
15	304	5	M	4.58	32	Ab+EI	51	None	None	Security
16	404	5	M	4.58	32	CCRF	51	Type II CRL, RCT	None	Security
17	104	6	M	2	17	UCF	40	RIC	None	Agility
18	304	7	F	1.2	31	CCF	12	RCT	None	Agility

*CCF, complicated crown fracture; UCF, uncomplicated crown fracture; CCRF, complicated crown-root fracture; Ab, abrasion; EI, enamel infraction; CL, Crown Lost*.

* *Replica*.

The mean time ±SD between preparation and cementation of the prosthodontic crowns was 10.5 ± 2.5 days. No adjustments were necessary as all crowns fit adequately.

**Figure 2:**
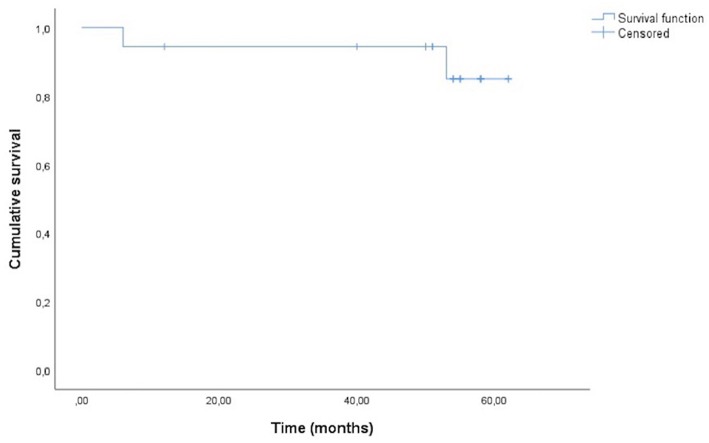
Survival curve of 18 CAD/CAM processed prosthodontic crowns in seven dogs.

Each dog resumed working activities successfully, with no signs of pain or discomfort noted by the owners/tutors. Follow up of all cases range from 12 to 62 months. Mean survival time for the crowns was 58.0 months (Figure 2). The mean ± SD follow-up period for all procedures, 21 crown cementations, including replicas was 47.4 ± 16.3 months (range: 1–62 months). Gingivitis, gingival recession, and gingival enlargement were not seen on the treated subject teeth during the follow-up period, and the owners reported no eating or behavior problems.

Out of the 18 full crowns placed, two mandibular canine teeth crowns were lost during the normal working activity of the dogs, for one tooth, the crown was lost twice (Table 1). In these cases, replicas were made using the previous scanned records, and they were cemented using the same procedure 6 and 10 days after, respectively. In the case where the crown was lost the second time, a new crown preparation was done adding axial grooves to the design. The second crown was still in place at the end of this study. Success rate of this case series was 88.9%, 2 events in a total of 18 crowns cemented.

**Figure 3:**
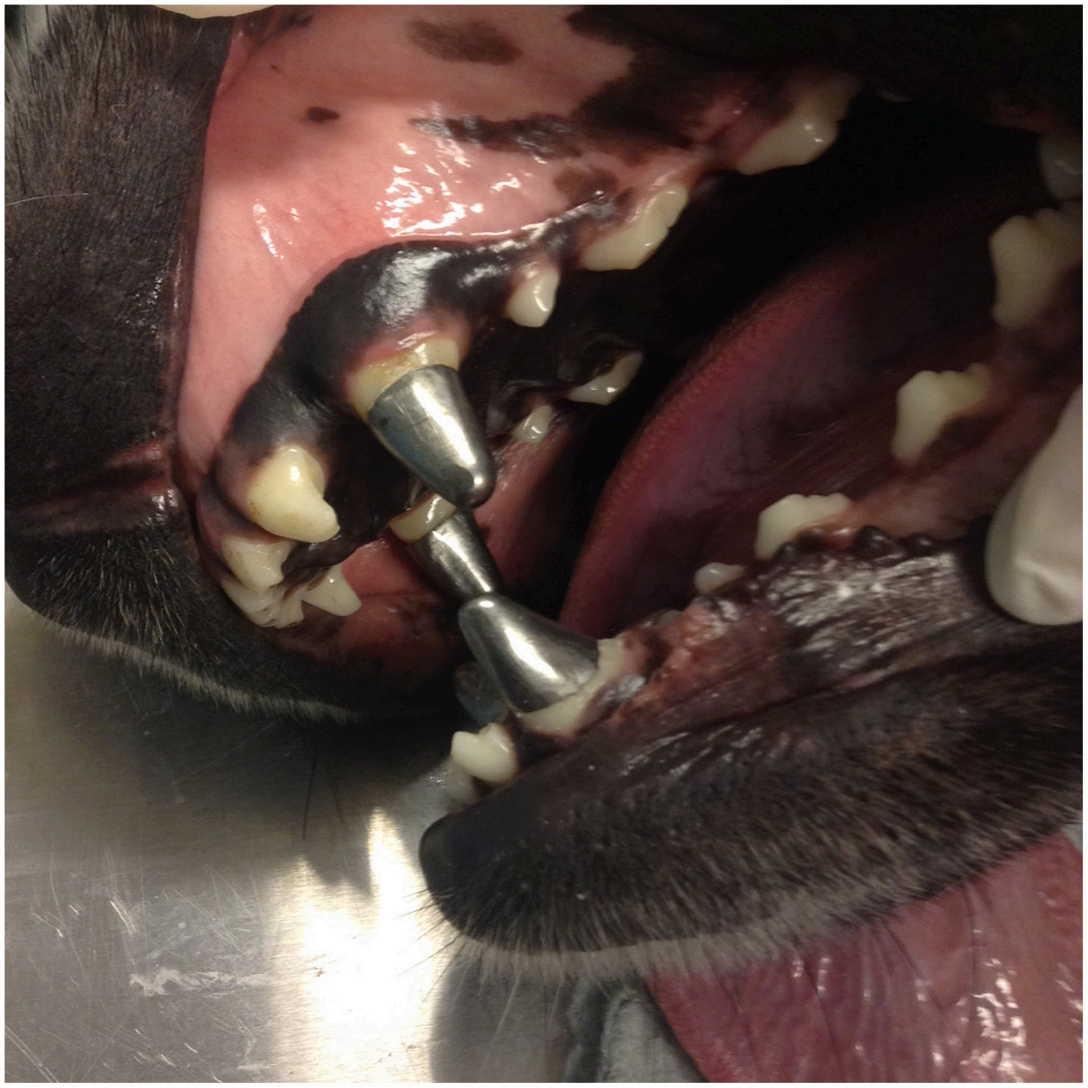
Roughness of the crown's surface and a loss of the characteristic metal shine registered at 1 year follow up.

At the time of the last recheck examination, the prosthodontic crowns showed no signs of toxicity or wear. However, after a few months, on average, a roughness of the crown's surface and a loss of the characteristic metal shine was observed (Figure 3). All the owners reported that they believed the crown was beneficial to the animal during its working activities.

## Discussion

The importance of restoration of fractured teeth has been widely described in the literature on human dentistry (13, 14). After endodontic treatment, there is a greater risk of injury to a non-vital tooth. More than the loss of dental substance there is the absence of nerve fibers in the pulp-dentin complex, which consequently limits sensitivity and pain reception (15, 16). In these situations (and especially in working dogs such as military dogs whose teeth are more exposed to wear and where having functioning teeth is of major importance for performance), crown therapy is recommended to protect the teeth and slow the wearing process (1).

In veterinary dentistry, strength and resistance are usually more important than aesthetics when it comes to selecting the best prosthodontic crown material. Therefore, cast metal restorations have consistently been described as the material of choice, providing the most strength compared to other materials (15, 17). Porcelain-fused to metal or ceramic crowns provide a similar appearance to normal teeth and have also been used in veterinary dentistry, but they are more prone to fracture in comparison to full-metal restorations (5, 18). Consequently, they are not recommended as prosthodontic crowns in working dogs, where the magnitude of bite forces achieved, during their daily working routine, can easily overlap porcelain resistance.

Titanium has physical and mechanical properties desirable not only for dental implants but also for prostheses (crowns and fixed dentures). These properties include low toxicity, very low allergenic potential, biocompatibility (which is associated with high corrosion resistance), strength and rigidity comparable to the properties of other noble and high noble alloys (19). The titanium's self-protective oxide film (titanium oxide) is an important feature of its biocompatibility and resistance to corrosion, and that of its alloys (20). In the current study, a titanium-aluminum-vanadium alloy was used for the crowns in preference to pure titanium due to the alloy's superior physical and mechanical properties (20). However, after a few months on average, a roughness of the crown's surface and a loss of the characteristic metal shine was observed. Such roughness could be attributable to the oxidative layer and possibly to wear or even corrosion. Some of the animals' work involves practicing activities that lead to abrasion. For example, in the case of Mondioring, during training and competitions, dogs occasionally bite a fabric sleeve protected by an internal steel mesh. Nevertheless, even in these dogs, no relevant abrasion or deformation of the material was noted in this study. Plaque accumulation was minimal, and there were no signs of gingivitis in any of the dogs in all follow up periods, beside issues due to the absence of daily home care. Finally, the roughness observed in the crowns may be attributable to corrosion, but this seems to be unlikely, as titanium is resistant to corrosion in the presence of oral fluids (20). The titanium oxide layer can be affected by external factors such as acidic pH and fluorides, leading to corrosion (20), but again, no home care was provided, and no fluoride pastes were applied for the dogs in the current study.

The authors recognize the limited number of clinical patients to compare results with previous studies on outcome of prosthodontic crowns. Regardless, its success rate, 88.9%, is comparable with two previous studies performed in canine teeth of working dogs (6, 8). Another study, performed in canine teeth, although not specified if performed all in working dogs, reported a superior success rate (21). This study reported a 9.7% crown failure due to adhesive/cohesive failure, which will be discussed further (21).

The geometrical configuration of the prepared clinical crown can influence success or failure of a prosthodontic crown. Several publications tried to contribute to the knowledge about the ideal configuration to improve the success of prosthodontic therapy in dogs (21–25). Tooth hight, diameter, convergence angle and other features such as axial grooves can be controlled by the operator in order to provide the best clinical crown configuration and surface area to increase its resistance and retention, ultimately affecting the longevity of the cementation. Some of the most relevant findings in these studies point toward an overall maximization of the clinical surface area. Indeed, an increase in surface area was related with an increase of force needed to unseat a crown and crowns with successful clinical outcomes had higher mean surface areas (23). These studies have not yet established a critical surface area for crowns made for canine teeth in the dog. On the other hand, height to diameter ratio of 1.6 can be a critical measurement used to assess the overall performance and cementation/adhesion failure rates (4, 21). In our study, for one tooth, the crown was lost twice. In all episodes, the clinical crown was not affected, suggesting a cementation/adhesion failure. However, faced with a second cementation/adhesion failure in the same tooth 1 month after the first episode, the authors believe that, in this case, the failure could be a consequence of insufficient surface area. For this reason, two axial grooves were added to increase surface area, since providing retention features such as axial grooves crown has been shown to increase crown retention (25). This prosthodontic crown is still in place today.

Concerning tooth preparation, a circumferential supragingival crown preparation is standard in veterinary dentistry. Although there are not as many published studies in veterinary dentistry, there is some evidence supporting this recommendation. The aim is to preserve the health of the periodontium, although it may not provide sufficient surface area for long-term success of the prosthodontic therapy (22).

One of the main advantages associated with CAD/CAM-generated dental restorations is an increase in quality, reproducibility and data storage, allowing improved precision, planning and efficiency and eventually reducing costs in human patients (10–12).

Previously, when replacement of lost crowns was required, the whole procedure (preparation, impression and cementing) was repeated, requiring the re-use of at least two sedation sessions under general anesthesia. Using CAD/CAM techniques, a new, perfectly fitting replica is produced, contrary to what can be obtained using standard laboratory methods. Even if stone models are stored the new crown will not be an exact replica of the original one. Such technology benefits the patient, saving unnecessary procedures and is more time- and cost-effective, which is of major importance in veterinary medicine.

As technology is rapidly evolving, new portable real-time scanners are now available for dentists, providing direct digital information, and eliminating the need for impressions and stone models. However, according to a recent review, there is not enough scientific information to support the use of intra-oral scanners in long-span restorations in human patients (26). There are still pitfalls to overcome before discarding dental impressions, namely because there is difficulty in obtaining information on deep margin lines when blood is present or when there is gingival inflammation or edema. Regardless, in veterinary patients, impressions cannot be replaced by the use of other technology in light of the current lack of knowledge about the comparative efficacy, despite the availability of new technology. The accuracy of impressions is based on the quality of the impression procedure, and the impressions are critical records that should be stored in case of the need for long-term restoration.

To the knowledge of the authors, there are only 3 published studies on the effectiveness of prosthodontic crowns in working dogs and no published data on the use of CAD/CAM technology for fabrication of prosthodontic crowns in a clinical veterinary dentistry setting (6, 8, 23). The results of this study suggest that full titanium alloy crown therapy is as successful as existing approaches for protecting teeth in working dogs, and CAD/CAM technology can also be considered in veterinary dentistry as a feasible option to improve the effectiveness of the prosthodontic crown fabrication process.

## Ethics Statement

This study, retrospective in nature, reviewed clinical cases managed using the highest standards of veterinary care, and consequently is exempted from ethical committee approval. All participants gave written informed consent for inclusion in the study.

## Author Contributions

LM, IG, JG, and MN: draft; JG and MN: critical revision; NL: substantial contribution; LM, IG, JG, NL, and MN: final approval of the version to be published.

### Conflict of Interest Statement

The authors declare that the research was conducted without any commercial or financial relationships that could be interpreted as a potential conflict of interest.

## References

[1] DuPont G Chapter 5: Pathologies of the dental hard tissues. In: Niemiec BA, editor. Small Animal Dental, Oral, and Maxillofacial Disease. London: Manson Publishing (2010). p. 128–56. 10.1201/b18171-6

[2] Golden AL Stoller M Harvey CE A survey of oral and dental diseases in dogs anesthetized at a veterinary hospital. J Am Anim Hosp Assoc. (1982) 18:891–99.

[3] Hamel L Le Brech C Besnier NJ Daculsi LG Measurement of biting-pulling strength developed on canine teeth in military dogs. J Vet Dent. (1997) 14:57–60.

[4] Soukup JW Collins C Ploeg HL. The influence of crown height to diameter ratio on the force to fracture of canine teeth in dogs. J Vet Dent. (2015) 32:155–63. 10.1177/089875641503200302 26638294 PMC5140095

[5] Bellows J Chapter 8: restorative equipment, materials, and techniques. In: Bellows J, editor. Small Animal Dental Equipment, Materials, and Techniques. Oxford: Blackwell Publishing (2004). p. 231–62.

[6] Van Foreest A Tijssens M Resultaten van adhesief gecementeerde metalen kronen op getraumatiseerde hoektanden bij werkhonden. [Adhesively cemented full-cast metal crowns on traumatized canine teeth in working dogs]. Tijdschr Diergeneeskd. (2007) 132:156–62. 17378487

[7] Hamilton CJ Ridgway RL. Dowel and core preparation and full gold coverage of maxillary canine teeth in a German Shepherd. Vet Med Small Anim Clin. (1976) 71:176–81. 766358

[8] Van Foreest AW Roeters FJM. Evaluation of the clinical performance and effectiveness of adhesively-bonded metal crowns on damaged teeth of working dogs over a two- to 52-month period. J Vet Dent. (1998) 15:13–20. 10.1177/089875649801500101 10518868

[9] Fink L Reiter AM. Assessment of 68 prosthodontic crowns in 41 pet and working dogs (2000–2012). J Vet Dent. (2015) 32:148–54. 10.1177/089875641503200301 26638293

[10] Beuer F Schweiger J Edelhoff D. Digital dentistry: an overview of recent developments for CAD/CAM generated restorations. Br Dent J. (2008) 204:505–11. 10.1038/sj.bdj.2008.350 18469768

[11] Ng J Ruse D Wyatt C. A comparison of the marginal fit of crowns fabricated with digital and conventional methods. J Prosthet Dent. (2014) 112:555–60. 10.1016/j.prosdent.2013.12.002 24630399

[12] Papadiochou S Pissiotis AL. Marginal adaptation and CAD-CAM technology: a systematic review of restorative material and fabrication techniques. J Prosthet Dent. (2018) 119:545–51. 10.1016/j.prosdent.2017.07.001 28967399

[13] Aquilino SA Caplan DJ. Relationship between crown placement and the survival of endodontically treated teeth. J Prosthet Dent. (2002) 87:256–63. 10.1067/mpr.2002.122014 11941351

[14] Nagasiri R Chitmongkolsuk S. Long term survival of endodontically treated molars without crown coverage: a retrospective cohort study. J Prosthet Dent. (2005) 93:164–70. 10.1016/j.prosdent.2004.11.001 15674228

[15] Coffman CR Visser L. Crown restoration of the endodontically treated tooth: literature review. J Vet Dent. (2007) 24:9–12. 10.1177/089875640702400102 17500483

[16] Visser C Coffman C Visser L Introduction to prosthodontics. In: 21st European Congress of Veterinary Dentistry: Book of Proceedings. Lisbon: European Veterinary Dental Society (2012).

[17] Costa LC Pegoraro LF Bonfante G Influence of different metal restoration bonded with resine on fracture resistance of endodontically treated maxillary premolars. J Prosthet Dent. (1997) 77:365–9. 10.1016/S0022-3913(97)70160-4 9104712

[18] Crowder SE. Care of metal crown restorations. J Vet Dent. (2010) 27:191–6. 10.1177/089875641002700312 21038836

[19] Acharya BL Nadiger R Shetty B Gururaj G Kuma KN Darshan DD. Brushing-induced surface roughness of two nickel based alloys and a titanium based alloy: a comparative study - *in vitro* study. J Int Oral Health. (2014) 6:36–49. 25083031 PMC4109236

[20] Jorge JR Barão VA Delben JA Faverani LP Queiroz TP Assunção WG. Titanium in dentistry: historical development, state of the art and future perspectives. J Indian Prosthodont Soc. (2012) 13:71–7. 10.1007/s13191-012-0190-1 24431713 PMC3634937

[21] Soukup JW Snyder CJ Karls TL Reihl J. Achievable convergence angle and the effect of preparation design on the clinical outcome of full veneer crowns in dogs. J Vet Dent. (2011) 28:72–82. 10.1177/089875641102800203 21916370 PMC3302665

[22] Soukup JW. Crown preparation design: an evidence-based review. J Vet Dent. (2013) 30:214–9. 10.1177/089875641303000403 24660306

[23] Riehl J Soukup J Collins C Siverling S Ploef HL Snyder CJ. Effect of preparation surface area on the clinical outcome of full veneer crowns in dogs. J Vet Dent. (2014) 31:22–5. 10.1177/089875641403100102 24902409

[24] Collins CJ Hetzel SJ Siverling S Ploeg HL Soukup JW. Quantitative comparison of mathematical models to measure surface area of canine teeth prepared to receive full veneer crowns in dogs. Front Vet Sci. (2015) 2:31. 10.3389/fvets.2015.00031 26664960 PMC4672189

[25] Goldschmidt S Collins CJ Hetzel S Ploeg H-L Soukup JW. The influence of axial grooves on dislodgment resistance of prosthetic metal crowns in canine teeth of dogs. J Vet Dent. (2016) 33:146–50. 10.1177/0898756416676558 28327073

[26] Mangano F Gandolfi A Luongo G Logozzo S. Intraoral scanners in dentistry: a review of the current literature. BMC Oral Health. (2017) 17:149. 10.1186/s12903-017-0442-x 29233132 PMC5727697

